# Inflammatory and Metabolic Responses to Different Resistance Training on Chronic Obstructive Pulmonary Disease: A Randomized Control Trial

**DOI:** 10.3389/fphys.2018.00262

**Published:** 2018-03-23

**Authors:** Bruna S. de Alencar Silva, Fábio S. Lira, Fabrício E. Rossi, Dionei Ramos, Juliana S. Uzeloto, Ana P. C. F. Freire, Fabiano F. de Lima, Luís A. Gobbo, Ercy M. C. Ramos

**Affiliations:** ^1^Skeletal Muscle Assessment Laboratory, Department of Physical Education, São Paulo State University (UNESP), Presidente Prudente, Brazil; ^2^Exercise and Immunometabolism Research Group, Department of Physical Education, São Paulo State University (UNESP), Presidente Prudente, Brazil; ^3^Immunometabolism of Skeletal Muscle and Exercise Research Group, Department of Physical Education, Federal University of Piaui, Teresina, Brazil; ^4^Department of Physical Therapy, São Paulo State University (UNESP), Presidente Prudente, Brazil

**Keywords:** chronic obstructive pulmonary disease, resistance training, inflammation, metabolism, interleukins

## Abstract

**Background:** Low-grade inflammation can be present in chronic obstructive pulmonary disease (COPD), which may affect the regulation of muscle protein and body metabolism. Regular exercise show improvement in muscle strength and dyspnea in patients with COPD, however, the response to training on inflammatory and metabolic disorders is unclear. In this study, we compared the effects of resistance training using weight machines and elastic resistance (bands and tubes) on the inflammatory and metabolic responses in patients with COPD.

**Methods:** Patients with COPD were randomized into three groups: elastic band group (EBG), elastic tube group (ETG), and weight machines equipment group (MG). EBG and ETG were analyzed together [elastic group (EG)]. The participants were evaluated for pulmonary function (spirometry), peripheral muscle strength (digital dynamometry), IL-6, TNF-α, IL-10, IL-15 (Immunoassay), glucose, triacylglycerol, total cholesterol, HDL-c, and albumin levels (Enzymatic colorimetric). Blood samples were collected to assess the acute and chronic exercise responses after 12 weeks of training protocol.

**Results:** The patient's mean age was 71.53 ± 6.97 years old. FEV_1_ (percent predicted) was 50.69 ± 16.67 and 45.40 ± 15.15% for EG and MG, respectively (*p* = 0.28). All groups increased muscle strength (*p* < 0.05) with no differences between groups. The acute response to exercise after 12 weeks of training showed improvement of inflammation when compared to baseline. Regarding the chronic effects, it was observed a decrease of all cytokines, except IL-10 (*p* < 0.05). After 12 weeks of training, the analysis of the metabolic profile presented a reduction in glucose concentration (*p* < 0.01), with no differences between groups (*p* = 0.30) and a decrease in triacylglycerol for the EG (*p* > 0.01).

**Conclusions:** Training with elastic resistances or conventional weight machines showed improvement of inflammation response after 12 weeks of training. Chronically, both training groups showed anti-inflammatory effects, with the EG showing a strong tendency to improve IL-10/TNF-α ratio and IL-10 levels.

Trial registration : RBR-6V9SJJ.

## Introduction

Chronic obstructive pulmonary disease (COPD) is a major public health problem due to its high prevalence and mortality rates (López-Campos et al., 2016). The complexity of the disease leads to deterioration of respiratory function, systemic involvement, and comorbidities (Brown and Martinez, 2016; Corlateanu et al., 2016).

Among the different systemic manifestations of COPD are disorders in the metabolic and inflammatory profile (Barnes, 2014, 2016). Systemic inflammation affects the regulation of muscle synthesis and consequently impairs muscle function (Cielen et al., 2014; Gea et al., 2015). Thus, individuals with COPD may present limitations to perform daily life activities and may show a lower quality of life (Maltais et al., 2014).

Patients with COPD involved in regular practice of exercise show improvement in muscle strength, mechanical efficiency, dyspnea, and quality of life (Lacasse et al., 2004; Garrod et al., 2007; McCarthy et al., 2015). However, the affects of exercise on inflammatory and metabolic parameters in patients with COPD is unclear.

Positive inflammatory responses of aerobic and resistance training performed in traditional weight machines have been reported in healthy (Forti et al., 2017), obese (Gerosa-Neto et al., 2016), and cachectic cancer adults (Teixeira et al., 2016). Also, the same responses have been investigated using alternative protocols, such as functional training (Neves et al., 2015) and elastic resistance (Schober-Halper et al., 2016) due to its low costs and easiness to perform, enhancing the access to treatment. However, it is not known if these types of training can induce a similar anti-inflammatory response when compared to traditional resistance training.

Elastic tools have already shown positive results in functional exercise capacity (Ramos et al., 2014) and muscular function (Nyberg et al., 2015) among patients with COPD. Furthermore, it is unclear the effectiveness of these interventions on the inflammatory and metabolic profile in this population.

Therefore, this study aimed to compare the effects of resistance training using weight machines and elastic resistance (bands and tubes) on the inflammatory and metabolic responses in individuals with COPD.

We hypothesized that elastic resistance training with bands or tubes could induce similar anti-inflammatory responses when compared to traditional strength training with weight machines.

## Methods

Clinically stable patients with moderate to severe COPD (GOLD, 2017) performed a 12-week training program in a specialized rehabilitation center. The sample size was defined based on a previous study (de Alencar Silva et al., 2016). To detect an improvement of 36.38 N in strength, standard deviation of 35.2 N, 80% of power, and significance level of 5%, it was necessary to include 15 subjects per group.

Patients were excluded if they were active smokers or presented unstable cardiac disease, musculoskeletal disorders that would prevent the implementation of the experimental protocol or performed another systematic exercise program. Subjects were also excluded if they presented disease exacerbations or complications that hindered the continuity of the treatment or that could influence the systemic inflammatory process.

All individuals were previously informed of the objectives and procedures of the study and signed a consent form to participate in the study (Declaration of Helsinki). All procedures were approved by the Ethics Research Committee of the São Paulo State University (CAAE: 46065315.7.0000.5402). Recruitment and follow-up of the individuals were conducted between 2015 and 2016 in a Rehabilitation Center in São Paulo, Brazil. This randomized trial was previously registered (RBR-6V9SJJ- http://www.ensaiosclinicos.gov.br/rg/RBR-6v9sjj/).

### Study design

The sample was randomly divided using numbered sealed brown envelopes into one of the three groups: elastic band training group (EBG), elastic tube training group (ETG), and training group with weight machines equipment (MG). An individual who was not related to the study performed this process. For data analysis, EBG and ETG were bound into the elastic group (EG). Although therapists and patients were not blinded for the interventions, data analysis was performed by an individual who was not involved with data collection.

**Figure 1:**
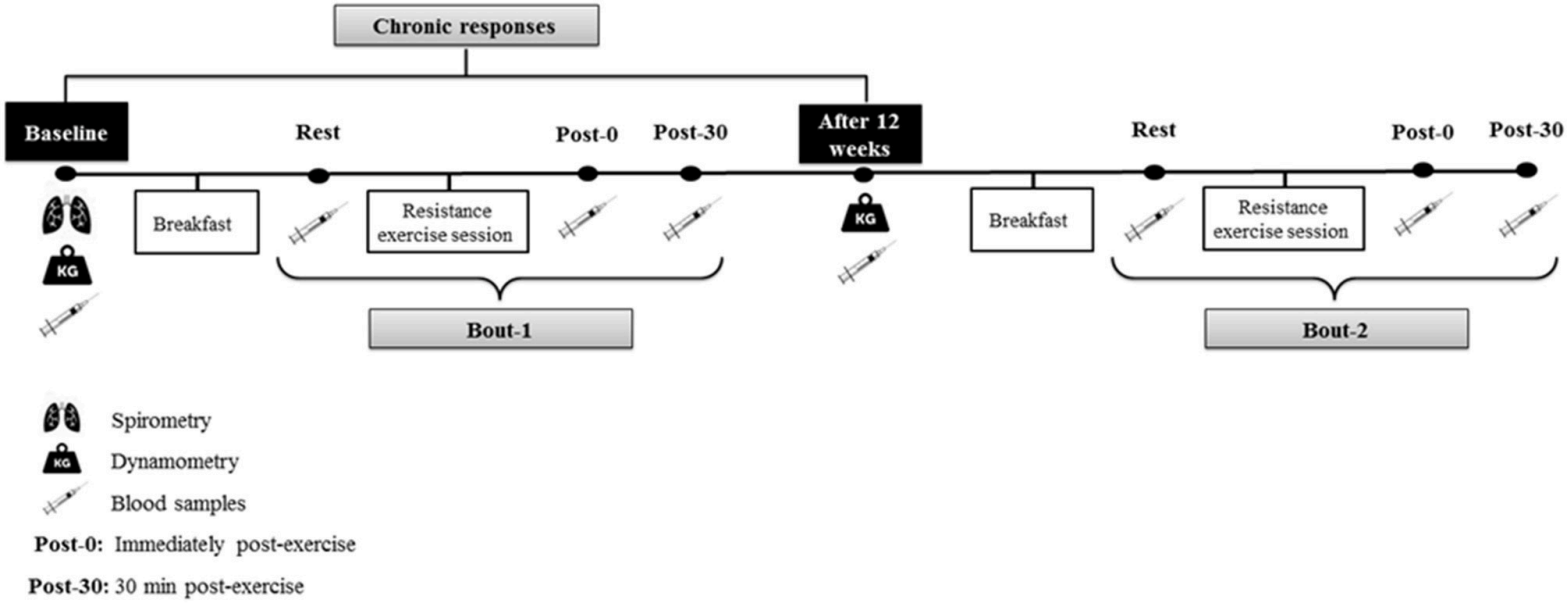
Acute and chronic blood analysis in baseline and after 12 weeks.

Patients initially underwent an assessment period performed individually and always at the same time of the day, in the morning, to avoid circadian variations. The first day of assessment consisted of anamnesis, personal identification, and spirometry test to confirm the diagnosis of COPD according to the American Thoracic Society and European Respiratory Society (Miller et al., 2005), with values of normality applied to the Brazilian population (Pereira et al., 2007). In the same week, patients underwent maximal peripheral muscle strength assessment by dynamometry (repeated after 12 weeks of training period). After 1 week, blood samples were collected in two different moments: (1) acute response to resistance training: acute session performed at baseline (Bout 1) and another acute session performed after 12 weeks of training protocol (Bout 2) and (2) chronic response to resistance exercise. For the first analysis, an acute training session (elastic bands, elastic tubes, or weight machine equipment) was performed at baseline (Bout 1) and was repeated after 12 weeks of training protocol (Bout 2), both with the same load (baseline load). The acute blood sample was collected in three different times: 1 h post-breakfast (Rest), immediately post-exercise (Post-0), and 30 min post- exercise (Post-30). For chronic analysis, blood sample was collected during fasting at baseline and after 12 weeks of training, according to what is shown in Figure 1.

### Dynamometry

Peripheral muscular strength was estimated by an electronic dynamometer (Power Din Standard, CEFISE, São Paulo, Brazil; elbow flexion and knee extension). Each test was performed three times for 8 s, with a 1-min interval. Peak force values were measured in newtons (N), and the highest values were recorded.

### Dietary intake

After blood sample collection, a standardized breakfast was offered, consisting of salted toast, processed UHT cheese (Ultra High Temperature), and strawberry yogurt. The number of macronutrients ingested was based on the total energy value, taking into account the height and total body mass by adopting a correction factor adjusted by the calorific value in relation to the physical condition assets, using the nutritional recommendation of 25% of the total energy value, referring to the total calories recommended for breakfast.

### Blood samples

The blood samples (15 ml) were collected in a room adapted for this purpose at the rehabilitation center and were immediately allocated into two 5 mL vacutainer tubes (Becton Dickinson, BD, Juiz de Fora, MG, Brazil) containing EDTA for plasma separation and into one 5 mL dry vacutainer tube for serum separation. The tubes were centrifuged at 2,510 × g for 15 min at 4°C, and plasma and serum samples were stored at −20°C until analysis. Cytokines IL-6 (range 2–200 pg/ml), IL-10 (range 2–300 pg/ml), TNF-α (range 4–500 pg/ml), and IL-15 (range 20–2,500 pg/ml) were assessed using human ELISA Ready-Set-Go kit (eBioscience® Vienna, Austria). The IL-10/TNF-α ratio was calculated by dividing IL-10(pg/ml) by TNF-α (pg/ml). Glucose (mg/dl), Triglycerides (mg/dl), Total cholesterol (mg/dl), and HDL cholesterol (mg/dl) were assessed using commercial kits (Labtest®, São Paulo, Brazil), and Albumin approximated measurement by the color change of Coomassie brilliant blue G-250 dye.

#### Acute collection

Initially, patients were instructed to fast for 12 h before the proposed evaluation. The blood collection was performed at 1 h post-dietary intake (Rest). After that, patients performed an exercise session (elastic bands, elastic tubes, or weight machine equipment) and then had the last two blood samples collected: immediately post-exercise session (Post-0) and after sitting for 30 min (Post-30) (Figure 1). After the completion of 12 weeks of training, the same procedures of blood collection were reproduced (Figure 1).

#### Chronic collection

For the chronic analysis, the patients were evaluated (fasting 12 h) at baseline and after 12 weeks of resistance training (Figure 1).

### Training protocol

The training protocol was performed for 12 weeks, three times per week, in sessions of ~60 min, for all groups (elastic resistances and traditional weight machines). The sessions started with stretching of the main muscle groups (shoulder flexion, elbow flexion, shoulder abduction, knee extension, and knee flexion) lasting 15–30 s each.

For EG group, training was performed in specially prepared chairs. The references of the elastic tubes used were: 200, 201, 202, 203, and 204 (Lemgruber, Brazil). Different tubes references have different thicknesses and, therefore, reflect different resistances (higher reference means greater resistance). For elastic bands (Therabanb®, USA), five different colors were used: yellow, red, green, blue, and black, respectively, from the highest to the lowest. Determinations of the length of the tubes and bands were performed according to the distance of the upper or lower limb of the patient to a fixed point in the chair, previously prepared for this purpose (de Alencar Silva et al., 2016).

For the MG we used traditional weight machines equipment (flexor and extensor chair and weight machines with washers for upper limbs; Supplementary Figure 1).

To perform all types of training, patients performed a protocol of a maximum number of repetitions in each set, representing that the imposed load was sufficient to promote only the established adaptations for each training week. During the first 2 weeks of training, the patient's imposed load was enough to complete two sets of 15 repetitions. For the fourth, fifth, and sixth weeks of training, the load was imposed to perform only three sets of 15 repetitions. Then, from the 7th to 9th week of training, the load was imposed to perform only three sets of 10 repetitions. Finally, in the last 3 weeks of training, the patients perform three sets of 15 repetitions. This protocol was adapted according to previous studies (de Alencar Silva et al., 2016).

### Statistical analysis

A Mauchly's test of sphericity was used to test this assumption, and a Greenhouse-Geisser correction was applied when necessary. A three-way analysis of variance was used to compare group (EG × MG), condition (baseline = bout 1 × after 12 weeks of training = bout 2), and time [Rest, immediately post-exercise (Post-0) and 30 min post-exercise (Post-30)] was conducted to verify the inflammatory and metabolic responses in the acute exercise sessions between groups.

For Chronic analysis, a Linear mixed model was used to compare EG and MG on the inflammatory, metabolic responses, and performance. Training intervention (elastic group and machine group) was included as the between-subject factor, time (Baseline and after 12 weeks) was included as the repeated within-subjects factor, time × intervention was included as the interaction, and the severity of COPD was included as a covariate. In cases where statistical interactions were present, a Bonferroni *Post-hoc* test was applied. Finally, delta values were conducted (Immediately post-exercise minus Rest; 30 min post-exercise minus Rest and 30 min post-exercise minus Immediately post-exercise, all values were divided by rest and multiplied by 100 = Δ%). Effect sizes for ANOVA were calculated using partial eta squared (η^2^), and classified according to Cohen (1988) using the following scale for interpretation: <0.2 [small]; 0.2 to <0.8 [moderate]; >0.8 [large] (Cohen, 1988). Data are reported as mean ± standard deviation. Statistical significance was set at *p* < 0.05. The data were analyzed using the Statistic software (version 10).

## Results

**Figure 2:**
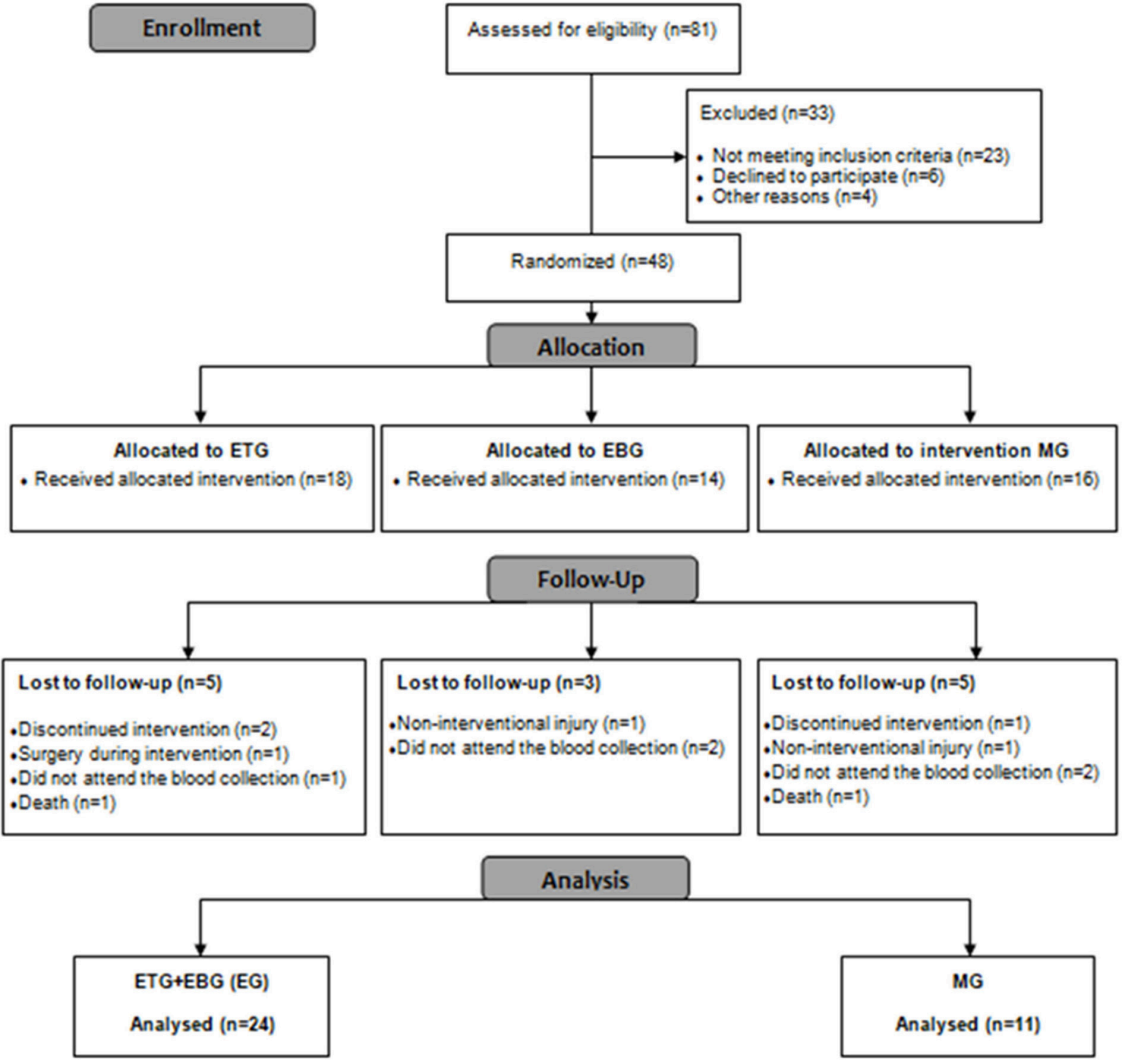
Flow diagram.

A complete overview of the study can be seen in Figure 2. Forty-eight patients with COPD were included and allocated into one of the groups. During the 12 weeks of training, 13 participants dropped out of the study [EG (*n* = 8)], and [MG (*n* = 5)] (a dropout rate of 27%) and, consequently, were excluded from the final analysis (Figure 2).

Table 1 presents the mean values of anthropometric data and spirometric indexes of the sample. There were no significant differences between groups at baseline (*p* > 0.05).

**Table 1:** Anthropometric data and spirometric indexes of the sample in baseline.

	**EG (*n* = 32)**	**MG (*n* = 16)**	***p***
**ANTHROPOMETRIC**			
Age (years)	69.35 ± 8.97	64.88 ± 11.17	0.13
BMI (Kg/m^2^)	26.38 ± 4.65	27.12 ± 5.00	0.60
**SPIROMETRIC**			
FVC % predict	72.48 ± 13.22	66.05 ± 13.97	0.12
FEV_1_ % predict	50.69 ± 16.67	45.40 ± 15.15	0.28
FEV_1_/ FVC	54.34 ± 12.86	55.01 ± 14.20	0.86

*BMI, body mass index; Kg, Kilograms; FVC, Forced vital capacity; Pred, predicted; FEV_1_, forced expiratory volume in the first second; EG, elastic group; MG, weight machine group. Data expressed as mean and standard deviation*.

### Acute responses

**Figure 3:**
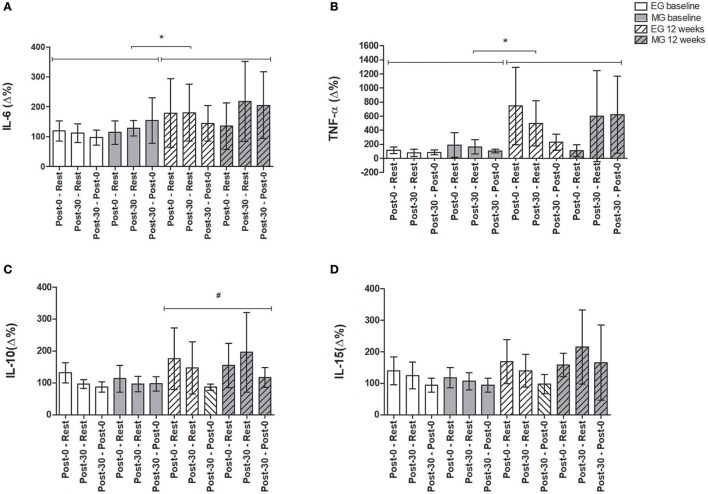
Analysis of inflammatory parameters (Δ%) in acute exercise session at baseline and 12 weeks after training protocol. Data expressed as mean and 95% confidence interval. **(A)** Interleukin 6, IL-6 (pg/mL); **(B)** Tumor necrosis factor-alfa, TNF-α (pg/mL); **(C)** Interleukin 10, IL-10 (pg/mL); **(D)** Interleukin 15, IL-15 (pg/mL). Post-0—Rest: difference between immediately post- exercise session and rest; Post-30—rest: difference between 30 min post- exercise and rest. Post-30—Post-0: difference between 30 min post- exercise and immediately post- exercise session. ^*^Significant differences between conditions between acute exercise at baseline and after 12 weeks (bout 1 vs. bout 2); ^#^Significant main effect of time (Rest, Post-0, and Post-30). In the comparisons between acute exercise at baseline and after 12 weeks of exercise training; EG: *n* = 24; MG: *n* = 11. Statistical test used: A Mauchly's test of sphericity was used to test this assumption, and a Greenhouse-Geisser correction was applied when necessary. A three-way analysis of variance were used to compare group (EG × MG), condition (baseline = bout 1 × after 12 weeks of training = bout 2) and time [Rest, immediately post-exercise (Post-0), and 30 min post- exercise (Post-30)] was conducted to verify the inflammatory and metabolic responses in the acute exercise sessions between groups. Finally, delta values was conducted (Immediately post-exercise minus Rest; 30 min post- exercise minus Rest and 30 min post- exercise minus Immediately post-exercise, all values were divided by rest and multiplied by 100 = Δ%).

Figure 3 shows the differences in the inflammatory profile (Δ%) for EG and MG submitted to an acute exercise session at baseline and another acute exercise session after 12 weeks of training.

For acute exercise session performed at baseline and acute exercise session after 12 weeks of training, there were no significant differences within time (Rest, Post-0, and Post-30). However, when compared the difference between acute exercise session at baseline and acute session after 12 weeks (bout 1 vs. bout 2), respectively, IL-6 (Δ%, *F* = 0.13, *p* = 0.04) and TNF-α (Δ%, *F* = 4.78, *p* = 0.04) increased significantly in the bout 2.

For IL-10 (Δ%) there was a main effect of time (*F* = 4.59, *p* = 0.03) with higher IL-10 at bout 2, but there was no significant interaction and difference between groups. For IL-15 (Δ%) there was no main effect of time (*F* = 3.33, *p* = 0.07) but there was a tendency to a significant increase at bout 2 (*F* = 3.74, *p* = 0.06) but no difference between group was observed (*p* > 0.05). The behavior of inflammatory profile (pg/ml) for EG and MG submitted an acute exercise session at baseline and after 12 weeks of training protocol can be observed in Supplementary e-Table 1.

### Chronic responses

For chronic analysis, there was an increase for peripheral muscle strength across time in the elbow flexion and knee extension, however, there were no significant differences between group (*p* > 0.05), both EG and MG induced a similar increased of strength after 12 weeks of training. Effect sizes were moderate in the EG (Elbow flexion = 0.53; Knee extension = 0.60) and moderate in the MG (Elbow flexion = 0.53; Knee extension = 0.65; Table 2).

**Table 2:** Effects after 12-week training of peripheral muscle strength between EG (*n* = 24) and MG (*n* = 11).

		**Baseline**	**12 weeks**	**Effect size**	**Delta post-pre (95%CI)**	***p***	**Effect**	***p***	***F***	**Effect size**
Elbow flexion (N)	EG	13.77 ± 5.32	16.78 ± 6.09	0.53	3.01 [0.83 to 5.18]		Group	0.09	3.02	0.08
						0.71	Time	<0.01	8.64	0.21
	MG	11.20 ± 4.87	13.54 ± 3.97	0.53	2.33 [−1.00 to 5.68]		Group × time	0.71	0.13	0.01
Knee extension (N)	EG	30.01 ± 11.08	37.21 ± 12.85	0.60	7.20 [3.30 to 11.19]		Group	0.05	4.27	0.11
						0.58	Time	<0.01	14.25	0.30
	MG	23.68 ± 8.72	29.04 ± 7.69	0.65	5.35 [−0.71 to 11.42]		Group × time	0.58	0.31	0.01

*N, newtons; EG, elastic group; MG, weight machine group. Data expressed as mean and standard deviation; Delta post-pre: difference between the initial and final evaluations of each group expressed as mean and 95% confidence interval*.

Table 3 shows chronic effects after 12 weeks of training on inflammatory profile between groups. There were no significant differences between groups at baseline (*p* > 0.05).

**Table 3:** Effects after 12-week training of inflammatory profile between EG (*n* = 24) and MG (*n* = 11).

		**Baseline**	**12 weeks**	**Effect size**	**Delta post-pre (95%CI)**	***p***	**Effect**	***p***	***F***	**Effect size**
IL-6 (pg/ml)	EG	2.41 ± 1.55	1.42 ± 1.04	0.76	−0.91 [−1.80 to 0.03]		Group	0.04	4.39	0.13
						0.48	Time	<0.01	10.55	0.27
	MG	3.90 ± 2.93	2.22 ± 1.80	0.71	−1.66 [−3.91 to 0.58]		Group × time	0.40	0.70	0.02
TNF-α (pg/ml)	EG	8.10 ± 10.83	4.17 ± 8.15	0.41	−3.45 [−5.88 to −1.03]		Group	0.31	1.05	0.03
						0.44	Time	<0.01	10.55	0.28
	MG	14.14 ± 20.18	6.90 ± 7.52	0.52	−8.15 [−18.11 to 1.80]		Group × time	0.34	0.94	0.03
IL-10 (pg/ml)	EG	1.64 ± 0.63	1.80 ± 1.47	0.15	−0.09 [−0.56 to 0.75]		Group	0.02	5.51	0.14
						0.06	Time	0.15	2.08	0.06
	MG	3.35 ± 2.60	2.30 ± 1.60	0.50	−1.05 [−2.19 to 0.09]		Group × time	0.06	3.03	0.16
IL-15 (pg/ml)	EG	57.73 ± 33.12	42.75 ± 32.91	0.86	−11.76 [−35.65 to 12.12]		Group	0.10	2.80	0.07
						0.69	Time	0.03	4.88	0.12
	MG	89.11 ± 100.30	65.80 ± 42.23	0.33	−25.73 [−73.08 to 21.62]		Group × time	0.63	0.23	<0.01
IL-10/TNF-α (pg/ml)	EG	0.42 ± 0.34	2.67 ± 4.01	1.03	2.05 [−0.11 to 4.22]		Group	0.17	1.16	0.08
						0.03	Time	0.10	2.79	0.09
	MG	0.67 ± 0.68	0.63 ± 0.53	0.07	−0.04 [−0.53 to 0.44]		Group × time	0.09	3.03	0.10

*IL-6, Interleukin-6; TNF-α, Tumor necrosis factor-alfa; IL-10, Interleukin-10; IL-15, Interleukin-15; IL-10/TNF-α, Interleukin-10/Tumor necrosis factor-alfa ratio. EG, elastic group; MG, weight machine group. Data expressed as mean and standard deviation; Delta post-pre: difference between the initial and final evaluations of each group expressed as mean and 95% confidence interval*.

For IL-6, there was a decrease after 12 weeks of training (*F* = 10.55, *p* < 0.01) and significant difference between groups (*F* = 4.39, *p* = 0.04) but no interaction was observed (*p* > 0.05). For TNF-α, there was a significant decrease statistically after 12 weeks of training (*F* = 10.55, *p* < 0.01), however, with no differences between the groups.

For IL-10 there was a lower reduction for EG in relation to MG (*F* = 5.51, *p* = 0.02). For IL-15, there was a decrease after 12 weeks of training (*F* = 4.88, *p* = 0.03) but there was no significant difference between group and interaction.

When analyzed IL-10/TNF-α ratio, there wasn't a main effect of time (*F* = 2.79, *p* = 0.10) and there was no significant interaction (*F* = 3.03, *p* = 0.09). Furthermore, delta analysis showed a greater IL-10/TNF-α ratio for EG compared to MG (*p* = 0.03).

Table 4 showed chronic effects after 12 weeks of training on metabolic profile between groups. There were no significant differences between groups at baseline (*p* > 0.05).

**Table 4:** Effects post 12-week training of metabolic profile between EG (*n* = 24) and MG (*n* = 11).

		**Baseline**	**12 weeks**	**Effect size**	**Delta post-pre(95%CI)**	***p***	**Effect**	***p***	***F***	**Effect size**
Glucose (mg/dL)	EG	90.45 ± 14.80	81.51 ± 17.46	0.55	−7.90 [−16.35 to 0.54]		Group	0,22	1.5	0.07
						0.59	Time	0.01	6.93	0.17
	MG	84.63 ± 26.95	71.13 ± 32.02	0.46	−12.74 [−34.62 to 9.13]		Group × time	0,59	0.28	<0.01
Total cholesterol (mg/dL)	EG	108.37 ± 25.30	104.58 ± 14.34	0.19	−5.58 [−15.68 to 4.52]		Group	0.63	0.23	<0.01
						0.88	Time	0,30	1.10	0.33
	MG	104.84 ± 38.42	99.86 ± 32.85	0.14	−3.32 [−22.55 to 15.90]		Group × time	0,88	0.02	<0.01
Triacylglycerol (mg/dL)	EG	154.17 ± 62.34	129.72 ± 40.32^*^	0.48	−23.87 [−43.66 to −4.08]		Group	0,97	<0.01	0.00
						<0.01	Time	0.66	0.18	<0.01
	MG	132.31 ± 43.60	150.34 ± 52.30	0.38	9.99 [−8.71 to 28.71]		Group × time	<0.01	8.25	<0.21
HDL cholesterol (mg/dL)	EG	58.44 ± 23.17	63.43 ± 17.25		0.40 [−8.81 to 9.62]		Group	0.18	183	0.05
						0.22	Time	0,92	<0.01	0.00
	MG	54.43 ± 21.90	50.12 ± 17.34	0.25	−3.20 [−11.38 to 4.97]		Group × time	0.22	1.53	0.04
Albumin (mg/ml)	EG	63.64 ± 16.83	62.56 ± 15.82	0.07	−1.27 [−11.69 to 9.15]		Group	0.52	0.40	0.01
						0.33	Time	0.24	1.43	0.04
	MG	71.88 ± 31.23	61.25 ± 15.40	0,46	−33.93 [−33.93 to 12.66]		Group × time	0.33	0.95	0.03
Total colesterol—HDL (mg/dl)	EG	50.70 ± 39.87	40.94 ± 25.81	0.30	−5.98 [−19.57 to 7.61]		Group	0.68	0.16	<0.01
						0.43	Time	0.36	0.82	0.02
	MG	50.41 ± 33.95	49.74 ± 24.50	0.02	−0.12 [−21.43 to 21.18]		Group × time	0,43	0.63	0.01

EG, elastic group; MG, weight machine group;

* *p < 0.05 in compression between baseline and 12 weeks in EG. Data expressed as mean and standard deviation; Delta post-pre: difference between the initial and final evaluations of each group expressed as mean and 95% confidence interval*.

Regarding the metabolic response, glucose decreased significantly after 12 weeks of training (*F* = 6.93, *p* = 0.01) but no differences between groups were observed. Triacylglycerol showed significant interaction (*F* = 8.25, *p* < 0.01). *Post-hoc* showed lower Triacylglycerol for EG in relation to MG (*p* = 0.008). For total cholesterol, HDL, albumin, and total cholesterol/HDL ratio, there were no significant differences across time, groups, and interaction.

All analyses of acute and chronic effects were also performed according to the severity of COPD according to GOLD (2017). However, no significant differences were found between categories (*Data not shown)*.

## Discussion

The main findings of the present study were: (a) in acute exercise session performed after 12 weeks of training, both groups showed a similar improvement of inflammation response when compared to baseline; (b) chronic effects showed that all training types were effective in decreasing pro-inflammatory cytokines and peripheral blood glucose levels. Also, the training with elastic resistance was effective to reduce triacylglycerol and sustained an anti-inflammatory profile, showing a tendency to improve IL-10/TNF-α ratio and IL-10 (Jung et al., 2008; Cabral-Santos et al., 2015; Harnish and Sabo, 2016). Thus, elastic resistance training may be an interesting strategy to induce anti-inflammatory effects in patients with COPD.

### Acute exercise effects

Exercises promotes changes in the inflammatory system profile and energy metabolism. IL-6 exert both effects pro- and anti-inflammatory, this specific function depend of the time and context where this cytokine is derived (Tanaka et al., 2014). IL-6 is produced in response to infections and tissue injuries, contributes to host defense through the stimulation of acute phase responses, hematopoiesis, and immune reactions. On the other hand, in acute exercise IL-6 is important for the anti-inflammatory response, since it stimulates the production of IL-10 and IL-1ra, which inhibits the action of nuclear factor kappa B and the synthesis of pro-inflammatory cytokines (Galic et al., 2010).

After exercising, the basal plasma concentration of IL-6, in healthy individuals, may increase up to 100-fold, and the combination of mode, intensity, contraction type, duration of the exercise determines the magnitude of the increase of exercise-induced plasma IL-6, with the peak level at the end of the exercise session (Pedersen and Febbraio, 2008).

In our study, an acute session of exercises with sedentary individuals showed a small influence on the production of systemic cytokines (Figure 3). It is known that IL-6 gene transcription is related to the quality of skeletal muscle contraction (Holmes et al., 2004), thus, musculoskeletal damage, such as muscular dysfunction present in COPD (Casaburi, 2000) could influence gene expression of IL-6.

IL-6 also performs an endocrine effect by increasing glucose uptake and fat oxidation (Pedersen, 2013). Scheele et al. (2012) demonstrated that, in insulin-resistant individuals, there is IL-6 resistance, as well as senescence of human skeletal muscle, may impair adaptive response to acute exercise by preventing a coordinated cytokine gene expression necessary to adequate muscle contractile function (Scheele et al., 2012). In this sense, the small inflammatory response found after an acute exercise session in sedentary individuals may indicate reduced endocrine activity of myocytes among COPD individuals in this study.

However, when these individuals are trained for 12 weeks, regardless of training type, elastic, or weight machines, the endocrine response of the skeletal muscles in producing these cytokines improves, mainly production of IL-6, which is the most apparent effector detected (Figure 3). Therefore, we could observe an efficient response after 12 weeks of training. Such adaptation could reflect important clinical benefits in these individuals due to the musculoskeletal dysfunction in COPD.

### Chronic exercise effects

Several studies have demonstrated a predominance of chronic low-grade inflammation in individuals with COPD (Joppa et al., 2016; Kang et al., 2016; Papi et al., 2016). The origin of this inflammation is still unknown, and it is related to a worse prognosis of COPD (Loprinzi and Walker, 2016). Strategies that soften it are strongly recommended, and exercise training may have an anti-inflammatory effect in heart failure (de Meirelles et al., 2014), diabetes (Karstoft and Pedersen, 2016), and cachexia (Lira et al., 2015), however, in COPD, the effects are still unclear.

Resistance training performed with elastic tools have shown to be an effective alternative to increasing muscular strength of individuals with COPD, similar to the use of weight machines (O'shea et al., 2004; Ramos et al., 2014; de Alencar Silva et al., 2016). As expected, our study showed a similar increase in peripheral muscle strength of knee flexion and extension after 12 weeks of training, with a similarity between elastic resistance and control group (weight machines).

Regarding the inflammatory and metabolic profile, our results showed that all training types were effective in decreasing pro-inflammatory cytokines and peripheral blood glucose, with an additional decrease in triacylglycerol in EG. The results corroborate the expected effects of resistance training (Petersen and Pedersen, 2005; Pedersen, 2012). However, comparisons should be performed with caution, since the age of the patients, type of exercise, intensity, frequency, and study design may result in different findings.

In patients with COPD, the anti-inflammatory effects of exercise training are controversial. In 2010, Van der Vlist et al. (van der Vlist and Janssen, 2010) conducted a literature review and concluded that endurance or resistance training showed beneficial effects on physical parameters in COPD, despite the absence of any decrease in inflammatory mediators. In 2016, Abd El-Kader et al. (2016) compared the response of inflammatory cytokines after aerobic vs. resistance training and showed that mean values of TNF-α, IL-2, IL-4, IL-6, and C-reactive protein (CRP) were significantly reduced in both groups after 12 weeks of exercise training.

The IL-10/TNF-α ratio was adopted as an indicator of inflammatory status, and low levels are associated with worse prognosis and greater susceptibility to co-morbidities (Kaur et al., 2006; Leonidou et al., 2007). Regarding this issue, the increase of this relation in patients with COPD is of extreme importance, since morbidity and mortality are related to increased systemic inflammation (Chen et al., 2012), which contributes to the pathogenesis of cardiovascular diseases (Sin and Man, 2003). The tendency of increased levels of the IL-10/TNF-α ratio in the elastic resistance training groups demonstrated an anti-inflammatory predominance in this type of training. The same result did not occur in the weight machines group, in which the levels of the interleukins did not change after training. A possible explanation for this finding in the elastic resistance training groups is the dynamics of the loads applied (elastic resistance and weight machines). Melchiorri and Rainoldi (2011) observed that the exercise performed with elastic resistance requires greater muscle activation, as well as the use of faster motor units compared to weight machines. The increased muscle activation may be one of the mechanisms explaining these findings since increased levels of IL-10/TNF-α were observed previously in high-intensity resistance training in trained individuals (Gerosa-Neto et al., 2016), as well as in exhaustive aerobic training in rats (Rosa Neto et al., 2009).

Concerning glucose metabolism, the increase of IL-6 in skeletal muscle contraction results in translocation of glucose transporter type 4 (GLUT 4) from intracellular compartments to the plasmatic membrane, resulting in glucose uptake (Carey et al., 2006). As a consequence, trained skeletal muscle will show adaptations, such as increased muscle glycogen, sensitivity to lipolysis, and oxidation of triacylglycerol (Pedersen, 2012). Thus, the muscles become less dependent on glucose and glycogen as a substrate during the exercise, which explains the decrease of glucose values at baseline found in this study.

Regarding the decrease of triacylglycerol only in the elastic resistance group, the characteristic of greater muscle activation may also have promoted a more efficient adaptation in this variable. Shepherd et al. showed that resistance and sprint interval training enhances oxidative muscle capacity and increases intramuscular triacylglycerol breakdown in type I and II fibers and its relation to the improvement of insulin sensitivity. This fact correlates with higher proteins lipid droplet-associated perilipin 2 and perilipin 5 expressions, which are involved in the adequate lipolytic control (Shepherd et al., 2013, 2014). In this sense, elastic resistance seems to be a better training protocol on reducing or preventing the risks of metabolic disease in patients with COPD.

### Strengths and limitations

As strengths of this study, we emphasize the importance of performing exercise training protocols using minimal, low-cost equipment, such as elastics resistances. It may be an alternative that facilitates the access to the treatment, mainly for home base rehabilitation and/or telerehabilitation (Scherr et al., 2009; Cranen and Groothuis-Oudshoorn, 2017). Therefore, it can be used as a strategy to improve service offering related to chronic lung disease. As limitations, it is possible to mention the non-standardization of loads between protocols of training, due to the difficulties of measurement in elastics resistance tools.

Thus, we confirm our hypothesis that elastic resistance training could induce similar anti-inflammatory effect when compared to traditional resistance training using weight machines. The findings are of great value for patients with COPD when considering the deleterious systemic effects that low-grade inflammatory profile can cause. In addition to the functional benefits already known regarding elastic training in patients with COPD, this study can add important information about the anti-inflammatory effect promoted by this type of training, which incorporates accessibility, versatility, may have a positive impact on the health promotion, and decrease public expenses with treatment.

In conclusion, this study demonstrated: (a) acute exercise session performed after 12 weeks of training showed improvement of inflammation response when compared to acute session performed at baseline, regardless of training type; (b) Chronically, all training types promoted an anti-inflammatory effect. Additionally, elastics training also showed a strong tendency to improve IL-10/TNF-α ratio, and IL-10 likely sustained an anti-inflammatory profile.

## Author contributions

BS, FSL, LG, ER, FR, and DR Provided concept, idea, and design of the work. JU, AF, and FFL Provided data collection. FR, BS, LG, and JU Provided data analysis. BS, FSL, LG, and FR Provided interpretation of data for the work. All authors contributed to the writing and revising of the manuscript, read, approved the final manuscript and agreed to be accountable for all aspects of the work in ensuring that questions related to the accuracy or integrity of any part of the work are appropriately investigated and resolved.

### Conflict of interest statement

The authors declare that the research was conducted in the absence of any commercial or financial relationships that could be construed as a potential conflict of interest.

## Supplementary material

Supplementary Figure 1 Representation of exercises performed in the three modalities of resistance training. **(A)** EBG; **(B)** ETG; **(C)** MG.

Supplementary e-Table 1 Analysis of inflammatory parameters (pg/ml) in acute exercise session at baseline and 12 weeks after training protocol.
