# Supplementary figures and images for: Inflammatory and Metabolic Responses to Different Resistance Training on Chronic Obstructive Pulmonary Disease: A Randomized Control Trial
Source: Front Physiol. 2018 Mar 23;9:262. doi: 10.3389/fphys.2018.00262 (PMC5877487; doi:10.3389/fphys.2018.00262)

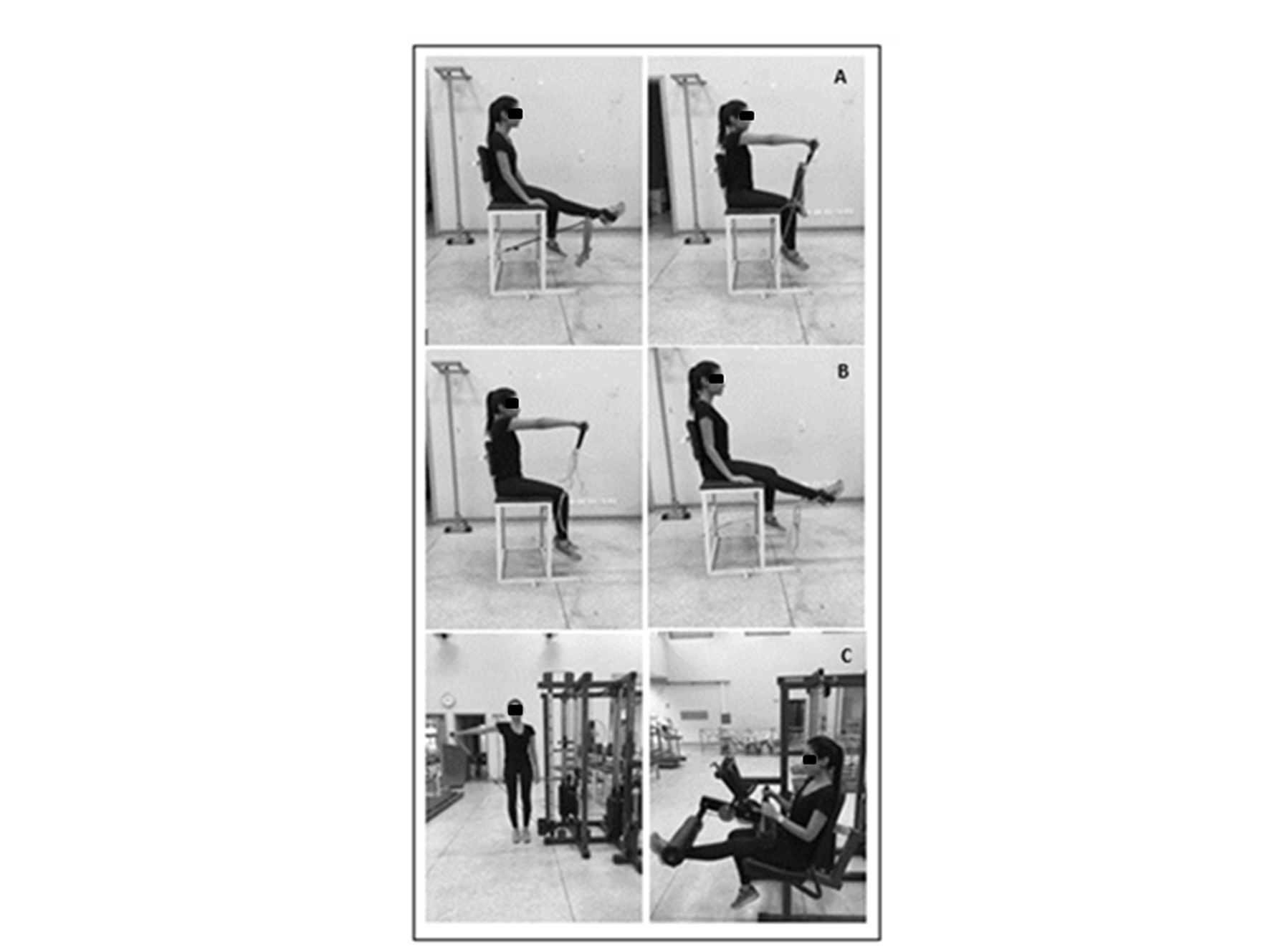

Supplement: Supplementary Figure 1 — Representation of exercises performed in the three modalities of resistance training. (A) EBG; (B) ETG; (C) MG. [file Image1.TIF]
