# Supplementary material for: Inflammatory and Metabolic Responses to Different Resistance Training on Chronic Obstructive Pulmonary Disease: A Randomized Control Trial
Source: Front Physiol. 2018 Mar 23;9:262. doi: 10.3389/fphys.2018.00262 (PMC5877487; doi:10.3389/fphys.2018.00262)
Supplement: Supplementary e-Table 1 — Analysis of inflammatory parameters (pg/ml) in acute exercise session at baseline and 12 weeks after training protocol. [file Table1.doc]

Supplementary Material

INFLAMMATORY AND METABOLIC RESPONSES OF DIFFERENT RESISTANCE TRAINING ON CHRONIC OBSTRUCTIVE PULMONARY DISEASE: A RANDOMIZED CONTROL TRIAL

Bruna Spolador de Alencar Silva, Fábio Santos Lira, Fabrício Eduardo Rossi, Dionei Ramos, Juliana Souza Uzeloto, Ana Paula Coelho Figueira Freire, Fabiano Francisco de Lima, Luís Alberto Gobbo, Ercy Mara Cipulo Ramos^*^

*** Correspondence:** Ercy mara Cipulo Ramos, ercy@bol.com.br

**Supplementary results e- Table 1**

e-Table 1 showed the differences in behavior of inflammatory profile (pg/ml) for EG and MG submitted an acute exercise session at baseline and after 12 weeks of training protocol.

For acute exercise session performed at baseline and acute exercise session after 12 weeks of training, IL-6 and IL-15 showed significant differences within time (F= 4.52, p=0.02; F= 4.74, p=0.02, respectively). In the comparisons between groups, IL-10 showed significant increase in EG compared to MG (F= 0.49, p=0.03). However, there were no significant differences between acute exercise session at baseline and acute session after 12 weeks (condition: bout 1 vs bout 2) in any variable.

**e-Table 1. Analysis of **inflammatory parameters (**pg/ml) **in** acute exercise session at baseline and 12 weeks after training protocol.**

|  | | |  | **EG** | **EG** | **MG** | **MG** | **Effect** | **p** | **F** | **Effect size** |  |
| --- | --- | --- | --- | --- | --- | --- | --- | --- | --- | --- | --- | --- |
|  | |  |  | **n=24** | **n=24** | **n=11** | **n=11** |  |  |  |  |  |
|  | | |  | **Bout 1** | **Bout 2** | **Bout 1** | **Bout 2** |  |  |  |  |  |
|  | | | **Rest** | 2.60±3.27 | 2.07±2.16 | 3.14±3.52 | 2.64±2.64 | **Group** | 0.35 | 0.89 | 0.03 |  |
| **IL-6 (pg/ml)** | | | **Post-0** | 2.86±3.41 | 2.71±3.47 | 2.98±2.29 | 3.34±3.65 | **Time** | 0.02 | 4.52 | 0.14 |  |
|  | | | **Post-30** | 2.84±3.84 | 2.44±2.25 | 4.04±4.39 | 3.68±2.53 | **Condition** | 0.34 | 0.91 | 0.03 |  |
|  | |  | **Rest** | 10.28±15.01 | 6.64±11.39 | 15.78±27.24 | 10.70±12.57 | **Group** | 0.67 | 0.17 | 0.01 |  |
| **TNF-α (pg/ml)** | | | **Post-0** | 9.86±17.16 | 10.48±18.96 | 14.07±20.49 | 8.05±13.97 | **Time** | 0.90 | 0.07 | 0.01 |  |
|  | | | **Post-30** | 7.63±14.75 | 7.73±8.55 | 17.94±27.84 | 10.71±7.96 | **Condition** | 0.08 | 3.19 | 0.12 |  |
|  | |  | **Rest** | 1.53±0.59 | 1.84±1.27 | 3.25±3.36 | 2.44±1.88 | **Group** | 0.03 | 0.49 | 0.13 |  |
| **IL-10 (pg/ml)** | | | **Post-0** | 1.85±0.89 | 2.09±1.04 | 3.13±2.72 | 2.85±2.13 | **Time** | 0.22 | 1.55 | 0.04 |  |
|  | | | **Post-30** | 1.40±0.59 | 1.76±0.92 | 3.29±3.85 | 2.97±2.21 | **Condition** | 0.75 | 0.10 | 0.01 |  |
|  | |  | **Rest** | 45.78±21.16 | 51.05±32.57 | 88.68±100.71 | 54.95±43.19 | **Group** | 0.06 | 3.89 | 0.11 |  |
| **IL-15 (pg/ml)** | | | **Post-0** | 54.75±25.58 | 66.30±45.95 | 87.07±77.12 | 82.75±62.96 | **Time** | 0.02 | 4.74 | 0.13 |  |
|  | | | **Post-30** | 47.51±28.02 | 55.29±41.47 | 81.98±76.66 | 93.15±51.54 | **Condition** | 0.98 | 0.01 | 0.01 |  |
|  | *IL-6: Interleukin-6; TNF-α: Tumor necrosis factor-alfa; IL-10: Interleukin-10; IL-15: Interleukin-15; IL-10/TNF-α ratoi: Interleukin-10/Tumor necrosis factor-alfa; EG= elastic group; MG= weight machine group; Condition: (baseline= bout 1 x after 12 weeks of training= bout 2) Bout 1: acute session performed at baseline; Bout 2: acute session performed after 12 weeks of training protocol;Time: (Rest, immediately post-exercise (Post-0) and 30 minutes post- exercise (Post-30)); Rest: immediately after the breakfast was offered; Post-0: immediately post-exercise session; Post-30: 30 minutes post- exercise session.* | | | | | | | | | | | |
